# Intrastriatally Infused Exogenous CDNF Is Endocytosed and Retrogradely Transported to Substantia Nigra

**DOI:** 10.1523/ENEURO.0128-16.2017

**Published:** 2017-02-20

**Authors:** Kert Mätlik, Helena Vihinen, Ali Bienemann, Jaan Palgi, Merja H. Voutilainen, Sigrid Booms, Maria Lindahl, Eija Jokitalo, Mart Saarma, Henri J. Huttunen, Mikko Airavaara, Urmas Arumäe

**Affiliations:** 1 Program in Developmental Biology, Institute of Biotechnology, University of Helsinki, 00014 Helsinki, Finland; 2 Electron Microscopy Unit, Institute of Biotechnology, University of Helsinki, 00014 Helsinki, Finland; 3 Functional Neurosurgery Research Group, Learning and Research Building, School of Clinical Sciences, Southmead Hospital, University of Bristol, Bristol BS10 5NB, United Kingdom; 4 Department of Gene Technology, Tallinn University of Technology, 12618 Tallinn, Estonia; 5 Herantis Pharma Plc, 00790 Helsinki, Finland; 6 Neuroscience Center, University of Helsinki, 00014 Helsinki, Finland

**Keywords:** CDNF, Parkinson’s disease

## Abstract

Cerebral dopamine neurotrophic factor (CDNF) protects the nigrostriatal dopaminergic (DA) neurons in rodent models of Parkinson’s disease and restores DA circuitry when delivered after these neurons have begun to degenerate. These DA neurons have been suggested to transport striatal CDNF retrogradely to the substantia nigra (SN). However, in cultured cells the binding and internalization of extracellular CDNF has not been reported. The first aim of this study was to examine the cellular localization and pharmacokinetic properties of recombinant human CDNF (rhCDNF) protein after its infusion into rat brain parenchyma. Second, we aimed to study whether the transport of rhCDNF from the striatum to the SN results from its retrograde transport via DA neurons or from its anterograde transport via striatal GABAergic projection neurons. We show that after intrastriatal infusion, rhCDNF diffuses rapidly and broadly, and is cleared with a half-life of 5.5 h. Confocal microscopy analysis of brain sections at 2 and 6 h after infusion of rhCDNF revealed its widespread unspecific internalization by cortical and striatal neurons, exhibiting different patterns of subcellular rhCDNF distribution. Electron microscopy analysis showed that rhCDNF is present inside the endosomes and multivesicular bodies. In addition, we present data that after intrastriatal infusion the rhCDNF found in the SN is almost exclusively localized to the DA neurons, thus showing that it is retrogradely transported.

## Significance Statement

The widespread and unspecific uptake of recombinant human cerebral dopamine neurotrophic factor (rhCDNF) after its delivery into brain parenchyma imply that therapeutically applied CDNF could directly influence a large number of cells in different brain areas. In addition, we show that infused rhCDNF is retrogradely transported from the striatum to the substantia nigra by the dopamine neurons that it rescues in rodent models of Parkinson’s disease. Along with the presented pharmacokinetic characteristics of rhCDNF after intrastriatal delivery, this information on the distribution of infused rhCDNF is important for the design of subsequent preclinical and clinical trials.

## Introduction

Cerebral dopamine (DA) neurotrophic factor (CDNF) and mesencephalic astrocyte-derived neurotrophic factor (MANF) are classified to form a novel family of neurotrophic factors (NTFs). Conventional NTFs, like members of the neurotrophin family, are small secreted proteins that potently stimulate neurite growth and promote the survival of those neurons that bear cell surface receptors specific for that particular NTF (for review, see Reichardt 2006; Saarma et al., 2011; Alsina et al., 2012). In this regard, MANF and CDNF are rather unconventional, even though their mode of action is poorly understood. Different from classic secreted factors, MANF and CDNF can protect the cells as intracellular proteins, as is evident from their antiapoptotic effect on cultured sympathetic neurons observed after intracellular overexpression, but not upon addition to culture medium (Hellman et al., 2011; Mätlik et al., 2015; L. Yu and U.A., unpublished observations). Both MANF and CDNF have been reported to localize to the endoplasmic reticulum (ER), having an N-terminal signal peptide as well as a C-terminal ER retention motif (Lindholm et al., 2007; Parkash et al., 2009; Glembotski et al., 2012; Oh-Hashi et al., 2012; Henderson et al., 2013; Fernández et al., 2014). The intracellular survival-promoting effect of MANF is dependent on its localization to the ER (Mätlik et al., 2015). The mechanism by which MANF and CDNF promote survival is not clear, but in the case of MANF it likely involves the regulation of ER stress. MANF is upregulated in response to ER stress-inducing stimuli (Mizobuchi et al., 2007; Apostolou et al., 2008), and there is chronic upregulation of unfolded protein response markers in the pancreata of MANF knock-out mice, which subsequently develop diabetes (Lindahl et al., 2014). CDNF has similar functional motives in its primary structure, suggesting that it could act similarly to MANF. However, the modes of action of CDNF are less well studied.

MANF and CDNF can be secreted (Lindholm et al., 2007, 2008; Tadimalla et al., 2008; Glembotski 2011; Henderson et al., 2013), suggesting that they may also act as extracellular factors. Curiously, and different from their response to conventional NTFs already at subnanomolar concentrations of the ligand, cultured neurons are not rescued from cell death by extracellularly applied MANF and CDNF (Lindholm et al., 2007; Hellman et al., 2011). The only exception is a recent study in which extracellular CDNF-protected cultured midbrain DA neurons only when applied at a concentration of ≥10 µM (Latge et al., 2015). Moreover, *in vitro* extracellular MANF and CDNF do not bind or enter any of the neuronal types tested so far (data not shown; Hellman et al., 2011). However, when delivered extracellularly into the striata of the 6-hydroxydopamine (6-OHDA)-treated rats, serving as a model of the dopamine deficiency seen in Parkinson’s disease (PD) patients, MANF and CDNF behave as bona fide NTFs, protecting dopaminergic neurons from degeneration. More importantly, in the neurorestoration experiments, when applied weeks after the neurotoxic lesion, they restore dopamine circuitry and lost neurological functions, thereby making them currently among the best candidates for disease-modifying treatment of PD (Lindholm et al., 2007; Voutilainen et al., 2009, 2011; Airavaara et al., 2012; Bäck et al., 2013; Ren et al., 2013). Furthermore, compared with the glial cell line-derived neurotrophic factor (GDNF), the best-studied protein with proven efficacy in animal models of PD, recombinant MANF, has been shown to have the advantage of relatively unhindered diffusion in brain tissue (Voutilainen et al., 2009; Barua et al., 2015). The effects of extracellularly applied MANF are not limited to DA neurons, because intracortical infusion of recombinant MANF protein protected brain tissue from ischemic injury (Airavaara et al., 2009; Yang et al., 2014). However, it is not clear whether MANF and CDNF exert their effects via activated cell surface receptors like classic NTFs (Henderson et al., 2013) or via some nonreceptor mechanisms, such as intracellular activity following internalization. Despite the potential of MANF and CDNF for the treatment of acute and chronic neurological diseases, the fate of these factors after intraparenchymal infusion into brain tissue has not been studied in detail. Therefore, we set out to characterize the distribution, clearance, and intracellular localization of recombinant human CDNF protein delivered into rat brain tissue.

## Materials and Methods

### Intracerebral infusion of recombinant human CDNF using a conventional metal needle

#### Stereotaxic surgery and immunohistochemistry

Experiments were approved by Finnish National Animal Experiment Board and performed according to the National Institutes of Health *Guidelines for the Care and Use of Laboratory Animals*. Male Wistar rats (200–500 g; RccHan:WIST; RRID: RGD_5508396) were anesthetized with isoflurane and 20 µg of recombinant human CDNF (rhCDNF; produced in a mammalian cell culture expression system; Biovian; *n* = 9 rats) or enhanced green fluorescent protein (GFP; catalog #4999-100, Biovision; *n* = 3) was infused intracerebrally into the striatum and cortex of the left hemisphere using a 10 μl Hamilton syringe with a 30 G blunt needle and the following stereotaxic coordinates: anteroposterior (A/P), +1.0; lateromedial (L/M), −2.7. A total of 4 µl of protein solution (5 µg/µl) was infused by first lowering the needle to dorsoventral (D/V) −5.0 (coordinates from skull surface) and lifting it by 1 mm (i.e., to D/V −4.0, −3.0, and −2.0) after each infused microliter (i.e., with 2 min intervals; infusion speed 0.5 µl/min). The infusion was started 30 s after lowering the needle, and the needle was kept in place for 4 min after the infusion. For producing a lesion of the nigrostriatal system, rats (*n* = 5) were first infused intrastriatally (A/P, +1.0; L/M, −2.7; D/V −5.0) with 5 μl of saline containing 20 μg of 6-OHDA (Sigma-Aldrich), using a speed of 1 μl/min. Three days later the rats received an intrastriatal infusion of 10 µg of rhCDNF close to the same site (A/P, +1.0; L/M, −2.7; D/V, −5.5) at a speed of 0.5 µl/min. Six naive rats received only the intrastriatal rhCDNF infusion and served as unlesioned controls (*n* = 6).

The animals were killed for transcardial perfusion at the time points of 2 h (*n* = 4 for rhCDNF; *n* = 2 for GFP), 6 h (*n* = 6 for rhCDNF; *n* = 1 for GFP; *n* = 5 for 6-OHDA plus rhCDNF), and 24 h (*n* = 2 for rhCDNF) after the end of infusion. The rats were first perfused with 200 ml of 0.9% NaCl solution, followed by 500 ml of freshly prepared 4% paraformaldehyde (PFA) solution in 1× PBS. The brains were dissected out and postfixed for 2–5 d in 4% PFA in PBS. Sections of *Cdnf^−/−^* mouse brains were obtained from mice that do not express any CDNF because of a full deletion of exons 2–4 of the *Cdnf* gene (M. Lindahl and M. Saarma, unpublished observations). After dehydration and clearing with xylene, the brains were embedded in paraffin wax and sectioned into 5-µm-thick coronal sections. The sections were deparaffinized and rehydrated through graded alcohol series. After heat-induced epitope retrieval in a microwave oven (10 min at 95°C in 5.5 mm citraconic anhydride solution, pH 7.4), the samples were allowed to cool down to room temperature and rinsed three times for 5 min with TBS buffer (50 mm Tris-HCl, pH 7.5, 150 mm NaCl) and two washes in TBS-T (50 mm Tris-HCl, pH 7.5, 150 mm NaCl, 0.1% Tween-20). H_2_O_2_ quenching of endogenous peroxidase activity was included for sections for which chromogenic staining was used. Primary antibodies were incubated in TBS-T at +4°C overnight, followed by three washes with TBS-T. For the detection of rhCDNF, we used the affinity-purified rabbit anti-CDNF antibody (DDV1; a gift from Dr. Johan Peränen, University of Helsinki, Finland) that is not specific to human CDNF but did not give any signal from the endogenous rat CDNF in the conditions we used here. The other primary antibodies used were rabbit anti-GFP (sc-8334, lot #H101, Santa Cruz Biotechnology; RRID: AB_641123), rabbit anti-tyrosine hydroxylase (TH; catalog #AB152, Millipore; RRID: AB_390204), mouse anti-NeuN (catalog #MAB377, Millipore; RRID: AB_2298772), mouse anti-TH (catalog #MAB318, Millipore; RRID: AB_10050306), and mouse anti-parvalbumin (catalog #P3088, Sigma-Aldrich; RRID: AB_477329), all used at 1:500 dilution. The localization pattern of infused rhCDNF in the striatum and cortex was confirmed with another anti-CDNF antibody derived from mouse (catalog #302-100, Icosagen AS; RRID: AB_11134475). However, this antibody was not sensitive enough for the detection of rhCDNF in the substantia nigra pars compacta (SNc). For immunofluorescent staining, Alexa Fluor 568 goat anti-rabbit IgG (H+L antibody, catalog #A-11011, Invitrogen; RRID: AB_2534078) and Alexa Fluor 488 goat anti-mouse IgG (H+L antibody, catalog #A-11001, Invitrogen; RRID: AB_2534069) were used. The nuclei were stained with DAPI (catalog #D9542, Sigma-Aldrich). The fluorescent images stacks were acquired using the TCS SP5 Confocal Microscope equipped with LAS AF 1.82 (Leica Microsystems). The objective was Leica HCX PL APO 63×/1.3 GLYC CORR CS (21°C). The lasers used were DPSS 561 nm/20 mW, OPSL 488 nm/270 mW, and diode 405 nm/50 mW, with the beam splitter QD 405/488/561/635. The images were analyzed with the Leica LAS AF software (RRID: SCR_013673).

For chromogenic staining, the Vectastain ABC kit (catalog #PK4001) and DAB Peroxidase Substrate Kit (catalog #SK-4100) were used for the detection of primary antibody (all from Vector Laboratories). After dehydration in graded alcohol series and clearing in xylene, the sections were mounted with DePeX mounting medium and imaged with a Pannoramic 250 Flash II Slide Scanner (3DHistech) using the combined 20×/0.8 numerical aperture objective. The contrast was increased slightly and equally in all images of immunostained sections to make the unstained regions more easily visible.

#### Immunoblotting

For analysis with Western blotting, Han-Wistar rats (RccHan:WIST; RRID: RGD_5508396) were anesthetized with isoflurane and 20 µg of rhCDNF in PBS (5 µg/µl) was infused into the striatum and cortex using a Hamilton syringe (coordinates and settings described above). Animals were killed either 15 min after the end of the infusion (*n* = 2) or 6 h after the infusion (*n* = 2). The brains were snap frozen in isopentane on dry ice. Rostral half of the infused hemisphere (down to A/P −2.0) was minced and homogenized in PBS containing 0.1% SDS, 1% NP40, 0.5% Na-deoxycholate, and protease inhibitors, using one complete mini-tablet (catalog #11836153001, Roche) per 12 ml of lysis buffer. The lysates was separated electrophoretically with a NuPAGE BisTrisMini gel (4–12%) and blotted onto PVDF membrane. Rabbit anti-CDNF antibody (1:1600, DDV1), mouse anti-alpha-tubulin (1:1600; catalog #T9026, Sigma-Aldrich; RRID: AB_477593), IRDye 680LT goat anti-mouse IgG (1:10,000; catalog #926-68020, LI-COR; RRID: AB_10706161), IRDye 800CW goat anti-rabbit IgG (1:10,000, catalog #926-32211, LI-COR; RRID: AB_621843), and the LI-COR Odyssey Scanner were used for detection.

#### Immunoelectron microscopy

For immunoelectron microscopy, a Han-Wistar rat was anesthetized with isoflurane and 20 µg of rhCDNF in PBS (5 µg/µl) was infused into the striatum and cortex using a Hamilton syringe (coordinates and settings described above). Two hours later, the animal was killed and perfused transcardially with 200 ml of 1× PBS, followed by 200 ml of fixative containing 2% PFA and 3.75% acrolein in 0.1 m Na-phosphate buffer (PB), pH 7.4, and 400 ml of 2% PFA in 0.1 m PB. A piece of the striatum was dissected out, postfixed for 2 h in 2% PFA and cut into 50-μm-thick sections using a vibratome. Tissue sections were rinsed with PBS, treated with 1% sodium borohydride for 30 min, and incubated in PBS containing 0.05% Triton X-100 for 50 min. After 30 min blocking with 20% goat serum, the sections were incubated for 48 h at +4°C in the presence of rabbit anti-CDNF antibody (1:250; DDV1) in PBS containing 5% goat serum. After PBS washes, the sections were incubated overnight at +4°C in 5% goat serum/PBS in the presence of goat anti-rabbit Fab' fragment conjugated to 1.4 nm gold (1:100; catalog #2004, Nanoprobes). Samples were postfixed with 1% glutaraldehyde (Sigma-Aldrich), quenched with 50 mm glycine in PB, rinsed in PB, and then in double-distilled water, followed by silver enhancement of the gold particles with the HQ Silver Kit (catalog #2012, Nanoprobes) for 3 min. Sections from the contralateral uninfused side were treated in an identical manner and served as a negative control. Samples were then osmicated with 1% reduced osmium tetroxide for 1 h and dehydrated through a series of ethanols and acetone prior to gradual infiltration into Epon (catalog #812, TAAB). The 60-nm-thin sections were cut from polymerized Epon blocks and imaged using a Jeol JEM-1400 electron microscope at 80 kV, equipped with an Orius SC 1000B (Gatan) CCD camera. The rest of the brain sample, including part of the striatum, was paraffin embedded and used to verify immunohistochemically that the brain sample in question displayed the expected pattern of anti-CDNF IR seen previously in other samples (data not shown).

### Intracerebral infusion of rhCDNF using a fused silica catheter

#### Stereotaxic surgery

The study was performed in accordance with University of Bristol animal care policies and with the authority of appropriate UK Home Office licenses. Adult male Wistar rats (225–270 g; Charles River) were anaesthetized with intraperitoneal ketamine (60 mg/kg; Ketaset, Pfizer Animal Health) and medetomidine (0.4 mg/kg; Dormitor, Pfizer), and placed in a stereotactic frame. Either 3.7 or 16 μg of rhCDNF in 5 µl of artificial CSF (Biovian) was infused bilaterally into the rat striatum (A/P, +0.75; M/L, ±3.0; D/V, −5.0) at a rate of 1 µl/min, using a custom-made catheter composed of fused silica. After the infusion, the catheter was left *in situ* for 5 min and then withdrawn at a speed of 1 mm/min. For immunohistochemistry (IHC) analysis, each rat received a high dose and a low dose of rhCDNF to the left and right striatum, respectively (*n* = 3 for each time point). For ELISA, the rats received injection with either the high or low dose of rhCDNF bilaterally into the striatum (*n* = 3 per dose for each time point).

#### Quantitative IHC

Rats were killed and underwent transcardial perfusion with 4% PFA at the time points of 20 min, 6 h, 24 h, 72 h, or 28 d after intrastriatal infusion of rhCDNF. Rat brains were fixed by immersion in freshly prepared 4% PFA in PBS for 4 h at room temperature. Samples were cryoprotected overnight in 15% sucrose in PBS, and were then transferred to cryomolds, embedded in OCT medium (Sakura), and were snap frozen in dry ice-cooled liquid isopentane. The blocks were used to prepare 16-μm-thick coronal cryosections on a Leica CM 3050S Cryotome. The sections were stained with rabbit monoclonal anti-CDNF (1:1000 dilution; Herantis Pharma) and donkey anti-rabbit IgG (H+L antibody), DyLight650 (1:500; catalog #ab96922, Abcam; RRID: AB_10680408). Mosaic images of coronal brain sections were recorded on a Zeiss AxioImager.Z1 microscope using high-aperture lenses and AxioVision 4.8 software (RRID: SCR_002677) driven by AxioCam MRm (10× lens, numerical aperture 0.45, 1× optocoupler). The target area (entire dorsal striatum in either hemisphere) was identified by drawing areas of interest (AOIs) on three images per rat. A second AOI excluded artifacts (wrinkles, air bubbles, tissue damage) interfering with the measurement and defined the area for quantitative image analysis. Immunoreactive objects were detected by thresholding and morphological filtering (size, shape). Once the parameters of the targeted objects had been defined in a test measurement, the quantitative image analysis ran automatically by using a macro so that the results are rater independent and fully reproducible. All measurements were made using ImageProPlus 6.2 software (Media Cybernetics).

#### rhCDNF ELISA

Rats were killed, and their brains were snap frozen at the time points of 20 min, 6 h, 24 h, 72 h, or 28 d after intrastriatal infusion of rhCDNF. The striatum and substantia nigra were dissected out, and a 10% w/v homogenate was prepared (in ice-cold PBS containing protease inhibitors) using TissueRuptor (Qiagen) followed by sonication of the suspensions (for 15 s, on ice). After a short centrifugation step (5000 g, 5 min, 4°C) the supernatants were used for rhCDNF detection using human CDNF Elisa Kit (catalog #K2-001-096, Icosagen AS) according to the manufacturer protocol. Pharmacokinetic variables, contrast at maximum response (C_max_), AUC_0_t_ (area under the curve) and half-life were determined using the Microsoft Excel software plug-in program pkf (J.I. Usansky, A. Desai, and D. Tang-Liu, Department of Pharmacokinetics and Drug Metabolism, Allergan, Irvine, CA).

## Results

### Localization and subcellular patterns of exogenous CDNF in the striatum and cortex

**Figure 1.**
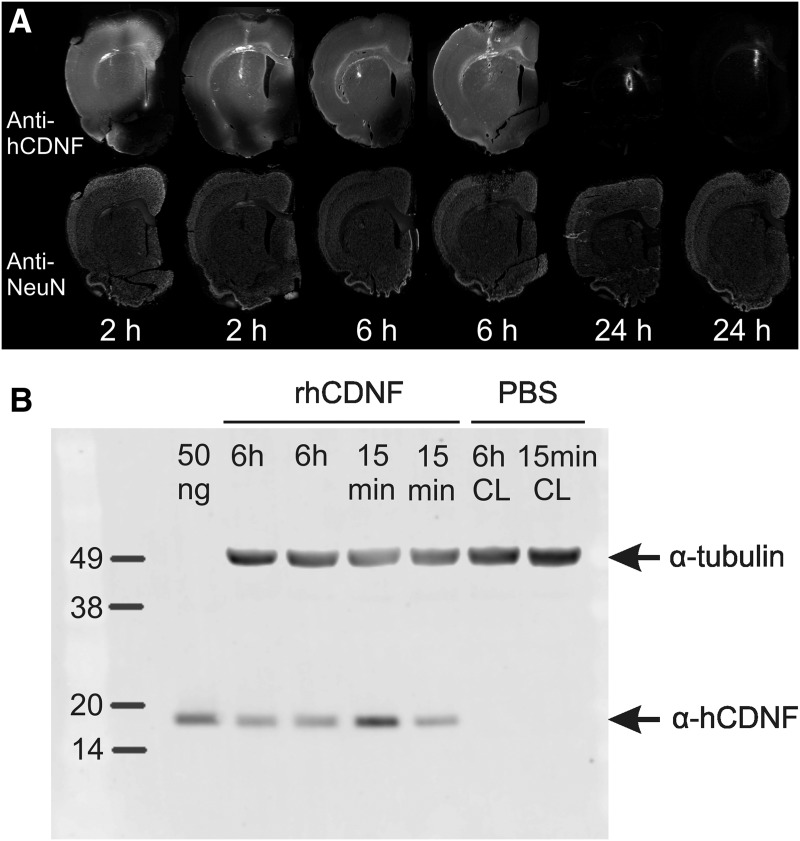
Stability of exogenous CDNF protein in rat brain. ***A***, Sections from rhCDNF-infused (20 µg) hemispheres were double stained with anti-CDNF and anti-NeuN antibodies, and each time point is represented by sections from two different animals. The time between rhCDNF infusion and perfusion of the animal is indicated below the images. A high level of anti-CDNF IR was detectable at positions up to 2 mm caudally from the infusion site, but there was very little spreading of rhCDNF to the contralateral uninfused hemisphere (data not shown). Scale bar, 2 mm. ***B***, Intactness of rhCDNF protein at 6 h after its infusion into rat brain. Lysates of rat brain at 15 min and 6 h after the infusion of rhCDNF were analyzed by immunoblotting with anti-CDNF antibody. Two animals were analyzed for each time point. Double staining with anti-α-tubulin antibody was used to ensure equal loading. Fifty nanograms of purified rhCDNF protein served as a positive control. CL, Contralateral uninfused hemisphere.

To get an estimate of the spreading and clearance of extracellularly applied CDNF in brain tissue, we first infused 20 µg of rhCDNF protein stereotaxically into the striatum and cortex of the left cerebral hemisphere of healthy young adult rats and killed the animals at 2, 6, or 24 h after infusion. After staining sections of their brains with anti-CDNF antibodies, a widespread strong anti-CDNF-immunoreactivity (IR) was detected in the infused hemisphere at 2 and 6 h after the infusion in all rats, extending almost over the entire hemisphere in the dorsoventral and mediolateral directions at the level of the infusion site (Fig. 1*A*). By 24 h after infusion, the majority of anti-CDNF IR had disappeared in all rats, except some granular IR at very close proximity of the infusion site (Fig. 1*A*). Since the anti-CDNF IR signal seen in stained sections could result from epitope-containing fragments of rhCDNF rather than the intact rhCDNF protein, we also analyzed CDNF-infused brains using Western blotting. The analysis showed that rhCDNF is still present in the brain as an intact protein at 6 h after the infusion, and no smaller fragments were detectable, even from the overexposed image (Fig. 1*B*; data not shown). This confirms that the signal seen in anti-CDNF-stained sections resulted mainly from the intact undegraded protein.

Next, we analyzed anti-CDNF-stained sections using confocal microscopy to study the intracellular localization of exogenous CDNF. To identify neurons, the sections were costained with antibodies to the neuronal marker NeuN. We concentrated on samples collected at 2 and 6 h after infusion (i.e., time points before most of the infused rhCDNF was cleared). Of note, as judged from the absence of anti-CDNF IR signal in uninfused and PBS-infused samples (below; data not shown), the levels of endogenous CDNF were too low to be detected with the anti-CDNF antibody raised against human CDNF.

**Figure 2.**
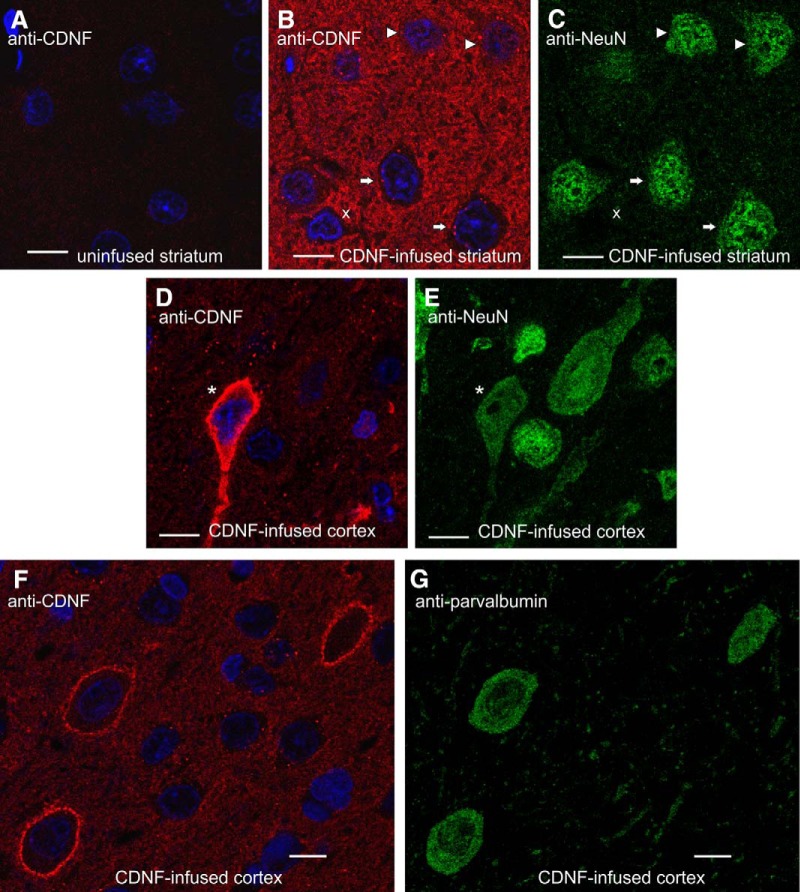
Subcellular localization patterns of infused rhCDNF in rat brain. Representative images of anti-CDNF IR at the 2 h time point, as observed by confocal fluorescence microscopy (*n* = 4). ***A–E***, Sections from the rhCDNF-infused (20 µg) or contralateral uninfused hemisphere were double stained with anti-CDNF antibodies (***A***, ***B***, ***D***) and anti-NeuN antibodies (***C***, ***E***). ***F***, ***G***, Sections from rhCDNF-infused hemisphere were double stained with anti-CDNF antibodies (***F***) and anti-parvalbumin antibodies (***G***). White arrows mark NeuN-positive cells that exhibit only the punctate CDNF-IR pattern, arrowheads mark cells that also exhibit diffuse anti-CDNF IR in the cytoplasm, the asterisk marks a neuron with strong cytoplasmic anti-CDNF IR, and a non-neuronal cell is marked with letter “x.” DAPI was used to stain nuclei (shown in blue). Scale bar, 10 µm.

Strong anti-CDNF IR was observed in both diffuse and granular pattern between the cell somata, whereas none of these patterns of anti-CDNF IR was seen on sections from the contralateral uninfused side (Fig. 2*A*,*B*). Four principal patterns of anti-CDNF immunoreactivity were observed inside the neurons (Fig. 2), with most of the striatal and cortical neurons being positive for at least one of these patterns. Quantification of these patterns is shown in Table 1. (1) Almost all neurons in the area where rhCDNF had spread contained strongly CDNF-positive puncta in the cytoplasm but not in the nuclei (Fig. 2*B*,*C*, white arrows). Our attempts to identify the CDNF-positive organelles with confocal microscopy failed as the antibodies to common markers of the endocytic compartments did not work on the brain sections (data not shown). (2) In ∼20% of the neurons, anti-CDNF IR was also seen as a weak diffuse pattern in the cytoplasm in addition to the puncta (Fig. 2*B*,*C*, white arrowheads). The nuclei of such neurons were not stained. (3) Some of the neurons and non-neuronal cells showed strong anti-CDNF IR throughout their cytoplasm. However, the location of these neurons was very variable from animal to animal (Fig. 2*D*,*E*, asterisk). These cells were most frequently apparent in the dorsal cortex (in 2 of 7 rats), lateral cortex (3 of 7 rats), and ventral striatum/ventral pallidum (in 3 of 7 rats). (4) Some of the neurons exhibited strong anti-CDNF staining at the boundaries of the cell (Fig. 2*F*). Almost all of these cells (65 of 67, as determined by an additional quantification) were parvalbumin positive (Fig. 2*G*) and are thus interneurons.

**Table 1:** Quantification of the observed anti-CDNF IR patterns at 2 h postinfusion from sections costained with antibodies to CDNF and NeuN

	Punctate	Weak diffuse (cytoplasmic)	Strong diffuse (cytoplasmic)	Strong (peripheral)
Cortex (70–130 cells scored per animal)	96.1 ± 3.4%	22.9 ± 13.3%	NQ	3.2 ± 2.0%
Caudate/putamen (69–107 cells scored per animal)	95.7 ± 3.7%	19.3 ± 8.2%	NQ	3.3 ± 1.7%

Randomly selected NeuN-positive cells in the caudate/putamen and cortex of the rhCDNF-infused hemisphere from four rats were scored on the basis of their anti-CDNF IR pattern. The number of cells matching the classification criteria is presented as a percentage of the total number of NeuN-positive cells scored (mean ± SD is shown, *n* = 4). NQ, Not quantified.

Some of the non-neuronal cells, defined as NeuN-negative cells with small nuclei, also had the punctate and diffuse patterns of anti-CDNF IR, although the slimness of the cytoplasm of these cells made quantification difficult (Fig. 2*B*,*C*, letter x).

These anti-CDNF IR patterns were revealed in both the striatum and cortex of all rats perfused either 2 or 6 h after infusion. The same anti-CDNF IR pattern was seen when we used another anti-CDNF antibody derived from another species (data not shown).

**Figure 3.**
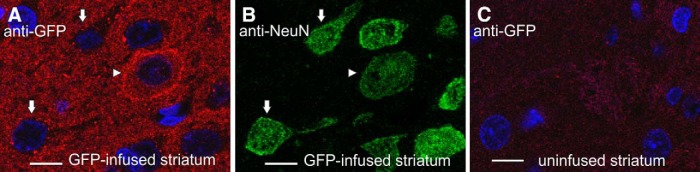
Subcellular patterns of anti-GFP immunoreactivity in rat brain 2 h after the infusion of recombinant GFP, as observed by confocal fluorescence microscopy. ***A–C***, Sections from the GFP-infused or contralateral uninfused hemisphere were double stained with anti-GFP antibodies (***A***, ***C***) and anti-NeuN antibodies (***B***). Arrows mark NeuN-positive cells with punctate anti-GFP IR pattern, and the arrowhead marks a cell that exhibits strong peripheral anti-GFP IR and diffuse anti-GFP IR in the cytoplasm. DAPI was used to stain nuclei (shown in blue). Scale bar, 10 µm.

To study whether the observed IR patterns were specific for rhCDNF, we also infused 20 µg of recombinant GFP to the striatum and cortex of the rats. We reasoned that a jellyfish protein should have no specific receptors or entrance mechanisms in the mammalian brain. To our surprise, we observed that anti-GFP antibody revealed all three IR patterns in the striatum and cortex at proportions approximately similar to those described above for rhCDNF (Fig. 3*A*,*B*), while there was only a very weak signal on sections through the contralateral uninfused hemisphere (Fig. 3*C*). Thus, exogenous CDNF and GFP may enter the neurons via the same or similar mechanism.

**Figure 4.**
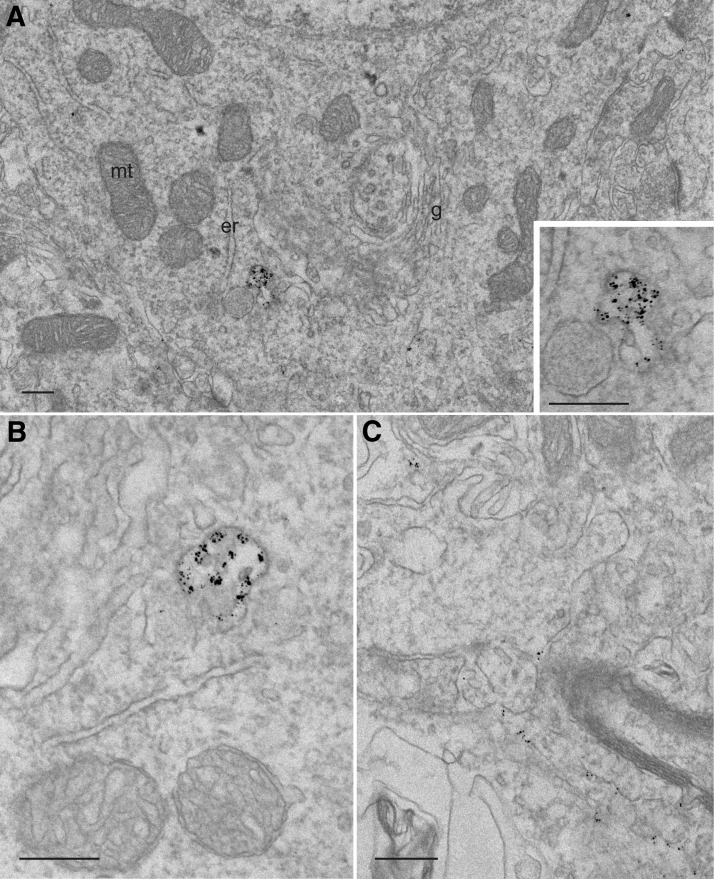
Immunoelectron microscopy of rhCDNF immunoreactivity in rat striatum 2 h after its intracerebral infusion. Shown are the representative images. ***A***, ***B***, Immunolabeling of infused rhCDNF can be seen in an early endosome (***A***) and in a multivesicular body (***B***). No labeling was observed in the ER, Golgi apparatus, or mitochondria. The inset in ***A*** shows a closeup of the anti-CDNF IR structure. ***C***, Anti-CDNF IR in the extracellular space. mt, Mitochondrion; er, ER; g, Golgi apparatus. Scale bar, 250 nm.

Our analysis using confocal microscopy could not reveal whether some of the infused rhCDNF ended up in the endoplasmic reticulum, the site where the endogenous or overexpressed CDNF protein is predicted to reside (Fernández et al., 2014) and possibly exerts its protective influence. In addition, our attempts to identify the CDNF-positive puncta using confocal microscopy failed, as antibodies against common markers of the endocytic vesicles did not work on paraffin-embedded brain sections (not shown). In order to resolve these questions, we turned to the pre-embedding immunoelectron microscopy. Staining of striatal samples revealed anti-CDNF IR of intracellular vesicle-like structures in the hemisphere where rhCDNF was infused (Fig. 4*A*,*B*), but never in the uninfused hemisphere (data not shown). Judged by morphology, the vesicles appeared to be endosomes, often having the characteristics of multivesicular bodies (Fig. 4*B*). Notably, a signal resulting from anti-CDNF IR was never seen in the endoplasmic reticulum, Golgi apparatus, mitochondria, or lysosomes (Fig. 4*A*; data not shown), but the amount of rhCDNF in these organelles could simply have been below the detection threshold for immunoelectron microscopy. In addition to the endosomes, anti-CDNF IR was sometimes seen in areas likely to be the extracellular space (Fig. 4*C*).

### CDNF is retrogradely transported by dopaminergic axons from the striatum to the substantia nigra

**Figure 5.**
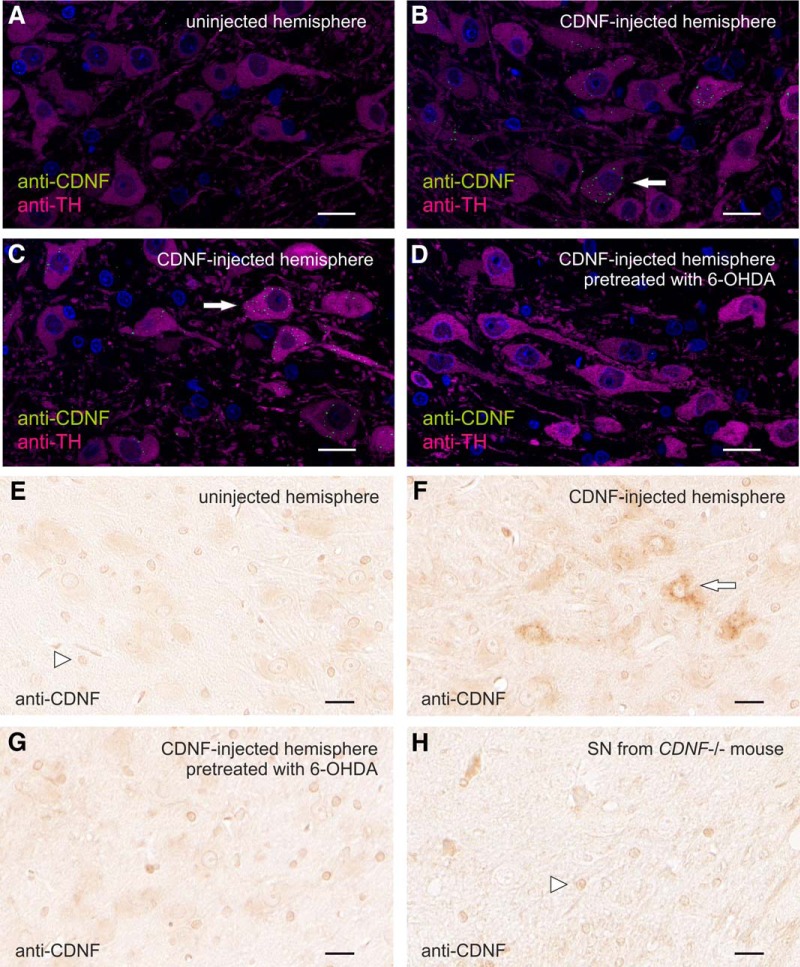
Localization of retrogradely transported rhCDNF in dopaminergic neurons of SNc. ***A–D***, Representative confocal microscopy images of anti-CDNF IR in SNc 6 h after infusion of 10 µg rhCDNF into the striatum (*n* = 5, 6). Sections from uninfused (***A***), rhCDNF-infused (***B***, ***C***), or 6-OHDA-treated rhCDNF-infused hemispheres (***D***) at the position of midbrain were double stained with anti-CDNF (green) and anti-TH (magenta) antibodies. ***E–H***, Chromogenic detection of anti-CDNF IR was used to analyze the SNc on sections from uninfused (***E***), rhCDNF-infused (***F***), or 6-OHDA-treated rhCDNF-infused hemispheres (***G***; *n* = 5–6). Sections from CDNF-deficient mice were used to reveal unspecific staining by the anti-CDNF antibody (***H***). Arrowheads mark the nuclei of non-neuronal cells nonspecifically binding the anti-CDNF antibody. Arrows mark some of the cells that contain punctate anti-CDNF IR. DAPI was used to stain nuclei in ***A–D*** (shown in blue). Scale bar, 20 µm.

In the 6-OHDA model of PD, the DA neurons, protected by intrastriatally infused rhCDNF, reside in the SNc (Lindholm et al., 2007; Voutilainen et al., 2011). However, the rhCDNF protein we infused did not diffuse extracellularly up to the SNc, as a high level of CDNF IR was detectable only at positions up to 2 mm caudally from the infusion site (data not shown). This indicates that, in order to reach the SNc, rhCDNF would have to undergo active intracellular transport. Indeed, some of the striatally infused radiolabeled rhCDNF was previously found to localize to the SN region, and this could be inhibited with an excess of unlabeled rhCDNF (Voutilainen et al., 2011). Unfortunately, the identity of the cells mediating this transport was not revealed. We therefore examined whether rhCDNF could undergo retrograde intracellular transport from the striatum to the SNc via the axons of the DA neurons. We cut sections through the SNc area of the brains of rats that had received intrastriatal rhCDNF infusion. These sections were costained with the antibodies against CDNF and TH, a marker for DA neurons, and analyzed with confocal microscopy (Fig. 5).

At 6 h after infusion, anti-CDNF-positive perinuclear puncta were clearly detected in the TH-positive cell bodies in the SNc, whereas very little anti-CDNF IR was seen outside the TH-positive area (Fig. 5*B*,*C*). A similar punctate pattern was seen in the SNc after chromogenic staining (Fig. 5*F*). Such CDNF-positive puncta were not observed in the SNc of the contralateral side (Fig. 5*A*,*E*) nor in the TH-positive neurons of PBS-infused hemisphere (data not shown), although rare random background dots were sometimes visible throughout the sections. After chromogenic detection of anti-CDNF IR, all samples displayed cross-reactivity of the anti-CDNF antibody with an epitope in the nuclei of non-neuronal cells. The unspecific nature of this anti-CDNF IR in the nuclei of non-neuronal cells was verified with the analysis of brain sections from CDNF-deficient mice, where this IR pattern could also be observed (Fig. 5*H*). Together, after striatal infusion, the rhCDNF transported to the SNc selectively ends up inside the TH-positive neurons, which is indicative of retrograde axonal transport by these cells.

A similar approach with the infused GFP protein did not reveal any GFP-positive vesicles in the TH-positive neurons of the SNc, even when larger amounts of protein were infused (40 µg; data not shown). This could reflect either the absence of retrograde GFP transport or the lower sensitivity of the anti-GFP immunodetection method.

**Figure 6.**
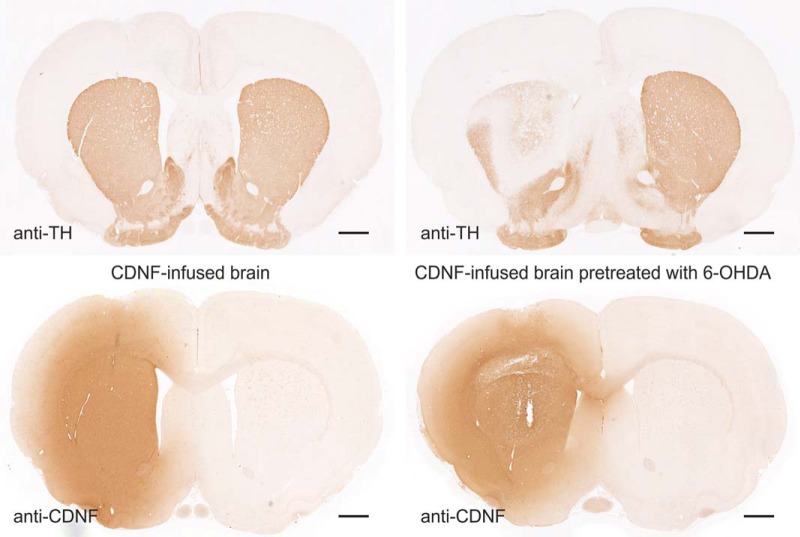
Loss of TH-positive nigrostriatal projections of DA neurons upon treatment with 6-OHDA. Images of anti-TH- or anti-CDNF-stained sections from brains collected 6 h after intrastriatal rhCDNF infusion (10 µg). On the right-hand side is the brain from a rat that had undergone intrastriatal infusion of 6-OHDA (20 µg) 3 d before. Scale bar, 1 mm.

To test whether the localization of rhCDNF to the SN is dependent on the striatal projections of DA neurons, we used intrastriatal 6-OHDA infusion to selectively damage the striatal DA nerve terminals (Fig. 6, top row), followed 3 d later by infusion of rhCDNF (Fig. 6, bottom row). As expected, 6-OHDA treatment led to a significant loss of TH-positive fibers in the dorsal striatum. Immunostaining of the SNc with anti-CDNF antibodies did not reveal CDNF-positive vesicles in the DA neurons (Fig. 5*D*,*G*), most probably due to the partial loss of their axons in the striatum. Thus, 6-OHDA-induced damage to the striatal projections of SNc DA neurons leads to a decrease in the retrograde transport of rhCDNF. We conclude that CDNF is taken up by the striatal TH-positive nerve terminals and is retrogradely transported to the cell bodies of DA neurons inside vesicular structures.

### The clearance of intrastriatally administered rhCDNF from rat striatum and substantia nigra

For a detailed analysis of the pharmacokinetics of intracerebrally infused rhCDNF in the striatum, we switched to a clinically more relevant administration method of using a catheter that is similar in design to the ones that have been used in clinical trials (Lewis et al., 2016). Two different rhCDNF doses, 3.7 and 16 μg, were selected based on previous studies of the 6-OHDA-induced rat model of PD (Lindholm et al., 2007). We infused rhCDNF protein into the rat striatum and killed the animals at different time points for analysis with two different methods: IHC and ELISA.

**Figure 7.**
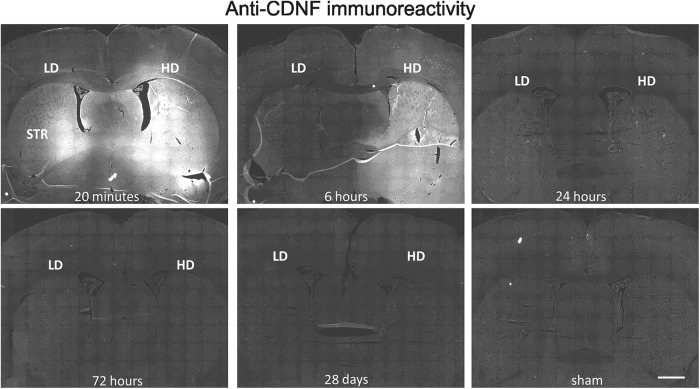
Spreading and clearance of rhCDNF after intrastriatal infusion. Representative images of anti-CDNF IR at different time points after infusion into striatum. Coronal sections from the level of A/P −0.3 are shown. LD, Low dose (3.7 µg) of rhCDNF; HD, high dose (16 µg) of rhCDNF; STR, striatum. Scale bar, 1 mm.

**Figure 8.**
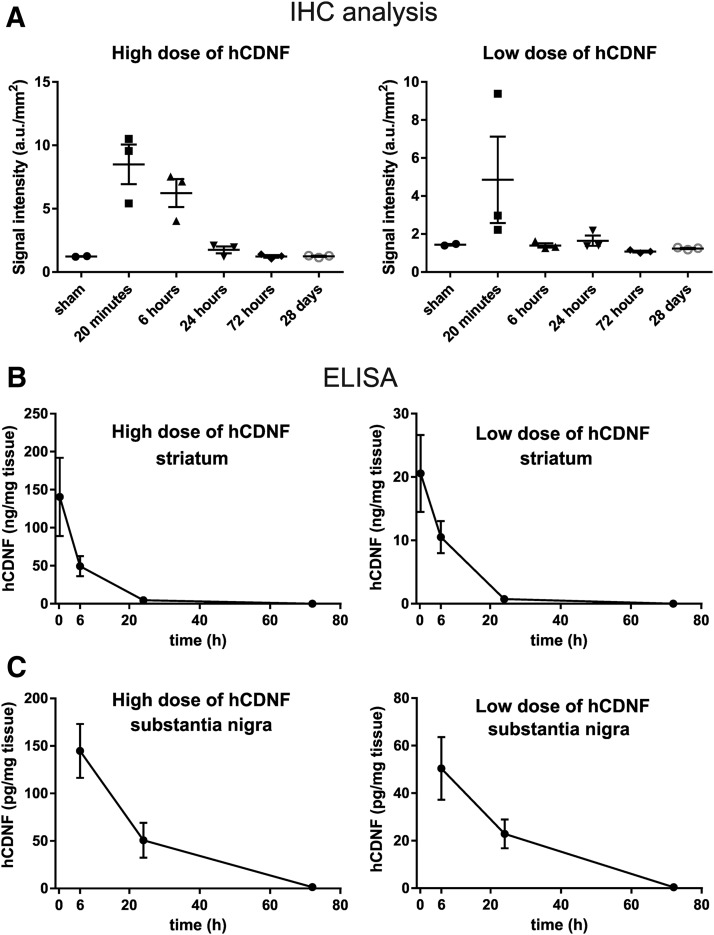
Quantitative analysis of rhCDNF clearance after intrastriatal infusion. ***A***, Quantitative analysis of CDNF immunofluorescence in the striatum. Data are shown separately for the left hemisphere, which received the high dose of 16 μg of rhCDNF, and the right hemisphere, which received the low dose of 3.7 μg of rhCDNF. Mean ± SEM values are shown; *n* = 3 hemispheres per time point. ***B***, Levels of rhCDNF in the striatum. ***C***, Levels of rhCDNF in the substantia nigra, as measured by ELISA. Mean ± SEM values are shown; *n* = 5-6 hemispheres per time point.

IHC analysis showed that already within 20 min the infused rhCDNF had spread in the dorsoventral and mediolateral directions to cover almost the whole striatum (within the A/P position covered by the coronal sections analyzed), reaching into the overlying corpus callosum and deeper layers of cerebral cortex (Fig. 7). In hemispheres where the high dose of rhCDNF was infused, strong anti-CDNF immunoreactivity persisted also at the 6 h time point, but at later time points the intensity of anti-CDNF immunoreactivity was not different from that measured from the sham-treated controls. This observation was verified by quantitative analysis of the anti-CDNF IR in the striatum (Fig. 8*A*).

Next, we used human CDNF-specific ELISA for measuring the amount of rhCDNF and calculating its pharmacokinetic characteristics after infusion into rat striatum. The level of rhCDNF in the striatum was high up to 6 h postinfusion, decreasing toward the 24 h time point and barely detectable after 72 h (Fig. 8*B*). By 28 d after infusion, full clearance had taken place (data not shown). The calculated pharmacokinetic characteristics are presented in Table 2. Both high dose and low dose resulted in a similar ∼5.5 h half-life of rhCDNF in striatum. The C_max_ and AUC_0_t_ for striatal hCDNF were relatively higher for the high-dose group, with the average high dose/low dose ratios for C_max_ and AUC_0_t_ being 8.4 and 6.0, respectively. In summary, both methods of intracerebral rhCDNF delivery showed that the protein spreads widely and a great majority of rhCDNF protein is cleared from rat striatum by 24 h after infusion.

**Table 2:** Pharmacokinetic data of rhCDNF in the striatum after intrastriatal infusion

	High dose	Low dose	Ratio
C_max_ (ng/mg)	140.6	20.6	6.8
AUC_0-t_ (µg/mg x min)	1.143	0.208	5.5
Half-life (h)	5.51	5.55	

We also measured the levels of rhCDNF in the SN at different time points after intrastriatal infusion. For both doses of rhCDNF infused, the protein was present in the SN at 6 h after delivery, with its local concentration in the SN being 200 to 300 times lower than in the striatum (Fig. 8C). The level of rhCDNF had become lower at the 24 h time point and almost undetectable by 72 h. Notably, while from 6 to 24 h after infusion the levels of nigral rhCDNF dropped ∼2.5-fold (for both of the doses; Fig. 8*C*), the apparent clearance seen during the same period in the striatum was much faster (decreasing ∼11- to 14-fold; Fig. 8*B*). In summary, after the transport of rhCDNF from the striatum to the SN, the protein is cleared from the SN within 3 d.

## Discussion

The main results of this study are that exogenous CDNF protein is (1) robustly internalized by CNS neurons and (2) retrogradely transported via the axons of dopaminergic neurons. We also report the rapid spreading of rhCDNF intraparenchymally infused into brain tissue and present its pharmacokinetic characteristics.

Intrastriatal delivery of rhCDNF has proven to be an effective treatment in rodent models of PD (Lindholm et al., 2007; Voutilainen et al., 2011; Airavaara et al., 2012), making it a good candidate for further preclinical and clinical studies. Importantly, the ability to distribute widely through the brain tissue is also one of the key factors to be taken into account when assessing the translational potential of intraparenchymal protein therapy. Therefore, knowledge of the distribution and clearance rate of CDNF after intrastriatal infusion is needed for the informed design of subsequent studies. For studying these characteristics of intrastriatally infused rhCDNF, we utilized a catheter similar in design to the one found to be effective in previous preclinical studies (White et al., 2011; Barua et al., 2012; Taylor et al., 2013). Our IHC analysis showed that rhCDNF spreads rapidly, already covering almost the whole rat striatum (at least in the dorsoventral and mediolateral directions) within 20 min after delivery. The immunoblot analysis of brain tissue immediately and at 6 h after delivery revealed only the full-size protein and no smaller fragments, implying that the widely distributed anti-CDNF immunoreactivity signal resulted from efficient spreading of the intact rhCDNF protein rather than from small epitope-containing fragments. This conclusion is further supported by our results from the ELISA, which is able to detect only the full-length human CDNF protein and not the separated N- or C-terminal domains (communication with the manufacturer Icosagen AS, Estonia). The efficient spreading of rhCDNF in brain tissue means that a rather large brain area, like the caudate-putamen of primates, can be covered with a small number of injections. A similar conclusion was reached for MANF in a study investigating its distribution after infusion into porcine putamen (Barua et al., 2015). The tissue half-life of infused rhCDNF in the striatum is 5.5 h, calculated from these ELISA measurements, and was found to be very similar for both doses of infused rhCDNF. Accordingly, the clearance of striatally infused rhCDNF was almost complete by the third day after delivery. The fact that by 24 h after infusion the level of rhCDNF in the striatum had decreased >25-fold was very well in line with the IHC analysis of a separate set of animals killed at the same time point. This wide spreading and clearance of rhCDNF was not particularly specific for the infusion method used, as a more conventional stereotaxic injection method into the rat striatum and cortex resulted in a largely similar spreading and clearance.

It is unknown whether rhCDNF is cleared mostly by efflux or local degradation. The calculated half-life of rhCDNF was well in line with that of dextran molecules and albumin (3–6 h), which is thought to be cleared by efflux from the parenchyma (Cserr et al., 1977; Groothuis et al., 2007; Iliff et al., 2012). However, since we noticed clear uptake of rhCDNF (and recombinant GFP) by almost all of the neurons in the area where rhCDNF had spread, local intracellular degradation is also likely to play a role. Compared with intrastriatally infused GDNF, the clearance of rhCDNF seems to be faster, as infusion of comparable amounts of GDNF resulted in ∼400-fold more of the protein still found in the striatum 3 d later (Hadaczek et al., 2010). This could result from tendency of GDNF to bind to the extracellular heparin and thus resist clearance by efflux from parenchyma (Piltonen et al., 2009).

By ELISA, rhCDNF could still be detected in the SN at 24 h after intrastriatal infusion but disappears within the next 2 d. The seemingly greater stability of rhCDNF in the SN, compared with striatum, most likely reflects the fact that in the striatum both extracellular and intracellular clearance mechanisms are operating, whereas only the latter acts in the SN, accompanied by a continuous influx of rhCDNF from the striatum.

Despite its relatively short persistence in rat brain, in the rat model of PD a single injection of rhCDNF, given 4 weeks after 6-OHDA administration, had a remarkably enduring effect at the level of behavior, lasting for up to 8 weeks (Lindholm et al., 2007). This has been interpreted as CDNF having a neurorestorative activity similar to GDNF and Neurturin, for instance. Indeed, the long-term changes of neural function following a rather short exposure to rhCDNF imply that this exposure leads to more permanent changes through its influence on gene expression, perhaps similarly to the way neurotrophic factors like GDNF influence gene expression through signal transduction cascades. How CDNF triggers the long-lasting survival-promoting effects in the brain remains to be studied.

Differently from other neurotrophic factors, the involvement of a cell surface receptor mediating the effects of CDNF and MANF has not been proven. Currently, the only evidence for the cell surface receptor-mediated mechanism is the reported association of some of the intracellular MANF with the cell surface. This association was dependent on the MANF ER retention signal, which is suggestive of binding to KDEL receptors present on the plasma membrane (Henderson et al., 2013). Remarkably, *in vitro* binding studies using radioiodinated MANF and CDNF have not shown binding or uptake in any of the cell types tested so far (Hellman et al., 2011; data not shown). This is in stark contrast to the effects these factors have in *in vivo* models. Therefore, we asked whether neurons in brain tissue internalize rhCDNF and where the internalized rhCDNF ends up inside the cells. We found that by 2 h after administration almost all of the striatal and cortical neurons surrounded by a sufficient concentration of rhCDNF had taken it up. However, the same was seen for infused recombinant GFP protein, which, in mammalian cells, presumably lacks a bona fide cell surface receptor. This argues that the uptake of rhCDNF cannot be mediated exclusively by a specific receptor. Although our immunolocalization data do not support the existence of CDNF-specific cell surface receptors, they do not exclude it either. Indeed, the signaling of the putative receptor could need only minute amounts of rhCDNF and could occur in parallel with the more abundant nonspecific uptake of rhCDNF.

Curiously, despite the apparent unspecificity of the uptake mechanism shared with GFP, all neurons did not exhibit the same pattern of subcellular localization. We do not know whether these patterns reflect different mechanisms of uptake or different routes of intracellular trafficking that follow a single mechanism of internalization (e.g., phagocytosis or macropinocytosis). Neither do we know which of these uptake patterns, if any, is relevant for the therapeutic effect of intrastriatally infused rhCDNF. Indeed, the patterns we describe simply could be indicative of intracellular degradation of foreign proteins by the brain. More studies are needed to solve this issue. Notably, the apparent unspecificity of these localization patterns suggests that similar uptake mechanisms could likely be seen for the CDNF homolog MANF, and some of them could be involved in the therapeutic effects MANF has in models of PD and cerebral ischemia (Airavaara et al., 2009; Voutilainen et al., 2009; Yang et al., 2014).

The predominant pattern of internalization, exhibited by almost all of the neurons, was a punctate one, pointing to an endosomal or lysosomal localization. Localization of rhCDNF into early endosomes and multivesicular bodies was verified with immunoelectron microscopy. However, lysosomes proved to be devoid of rhCDNF, due either to fast rhCDNF degradation and epitope destruction inside the lysosomes or to the escape of internalized rhCDNF from the lysosomal degradation pathway.

The other patterns of rhCDNF and GFP localization were seen in only a fraction of the neurons. The strong cytoplasmic staining appeared to be especially variable as it was seen in only some of the animals, with the location of these neurons varying from one rat to another. The weak diffuse cytoplasmic pattern of anti-CDNF IR was seen more consistently in approximately a quarter of neurons throughout the striatum and cortex. In neither of these cases could we assign these patterns of uptake to a specific subpopulation of neurons (e.g., interneurons or projection neurons). Nevertheless, both of these uptake patterns that we describe as cytoplasmic could very well represent proteins localized to the endoplasmic reticulum. Notably, the strong punctate and the weak diffuse staining patterns were not mutually exclusive, making it conceivable that rhCDNF could reach the ER via early endosomes. The possibility that at least some of the administered rhCDNF could end up in the ER is worth considering since this is the organelle where most of the endogenous CDNF is thought to be localized and where its homolog MANF needs to be in order to exert its neuroprotective function *in vitro* (Mätlik et al., 2015). Unfortunately, the limits of resolution in our confocal microscopy analysis prevented us from assigning either of the cytoplasmic immunoreactivity patterns to the ER. According to the immunoelectron microscopy analysis, the ER was lacking rhCDNF, as were the Golgi apparatus and mitochondria, but it could be that the amount of rhCDNF in organelles other than endocytic vesicles was simply below the detection threshold for immunoelectron microscopy. As an additional caveat, the rare and variable appearance of neurons with strong cytoplasmic staining could mean that we were unable to “catch” these cells in our immunoelectron microscopic analysis.

The most distinctive of all four subcellular localization patterns was the apparent accumulation of rhCDNF and GFP at the boundaries of parvalbumin-positive GABAergic interneurons. This most likely represents the nonspecific accumulation of rhCDNF and GFP to the perineuronal nets (PNNs) of extracellular matrix, which is reminiscent of the *Wisteria floribunda* agglutinin staining that is used to mark the PNNs that surround these neurons (Kwok et al., 2011).

Even though indirect effects of rhCDNF through cells of the striatum cannot be excluded, the DA neurons of SNc are still considered to be the direct targets of striatally administered rhCDNF in the PD models. Striatally infused radioiodinated CDNF has earlier been found in the SNc by radioautography and radioactivity counting (Voutilainen et al., 2011). Here, we used immunohistochemical methods to demonstrate the uptake of striatal rhCDNF by DA neurons on the cellular level. We show here for the first time that striatally administered rhCDNF does indeed end up in the somata of TH-positive DA neurons in SNc, which is indicative of retrograde axonal transport of rhCDNF from the striatum. The perinuclear pattern of anti-CDNF IR and its presence in some but not all TH-positive neurons of SNc was very similar to the patterns previously reported for striatally administered GDNF (Tomac et al., 1995). Internalization of the infused rhCDNF by almost all of the surrounding striatal neurons, including the projection neurons, suggests that it could also be transported anterogradely. However, there was almost no anti-CDNF IR signal outside the TH-positive area of SNc. In addition, the detection of rhCDNF in the SNc was dependent on the intactness of striatal nerve endings of DA neurons, as rhCDNF was undetectable when rats were infused intrastriatally with 20 µg of 6-OHDA. These observations speak against prominent anterograde axonal transport by SNc-innervating striatal GABAergic projection neurons, thereby ruling out the other possible explanation of the previously published autoradiography experiments (Voutilainen et al., 2011). The function and fate of this retrogradely transported rhCDNF is not known, but it is tempting to speculate that this pool of rhCDNF prevents the death of DA neurons in those models of PD where the nigrostriatal tract is still intact at the time of rhCDNF delivery (Lindholm et al., 2007; Airavaara et al., 2012). Retrogradely transported rhCDNF could perhaps act similarly to neurotrophic factors known to be transported in signaling endosomes (Harrington and Ginty, 2013). Alternatively, despite the low levels of CDNF that reached the SN, it could be that some of the transported rhCDNF ends up in the ER where it exerts its prosurvival influence, similarly to intracellularly overexpressed MANF (see above).

In the neurorestoration experiments, rhCDNF protected and repaired the nigrostriatal DA system that was already severely damaged by 6-OHDA (Lindholm et al., 2007; Voutilainen et al., 2011). While we cannot conclude that in 6-OHDA-treated brains the nigrostriatal transport of rhCDNF is completely absent, in our experiments a pretreatment with 6-OHDA leads to an apparent decrease in the retrograde transport of rhCDNF to the somata of DA neurons. Thus, the importance of CDNF retrograde transport in restoring the 6-OHDA-damaged DA system remains to be studied. Other possible mechanisms include local action at the DA axons not involving the cell bodies or signaling through the putative cell surface receptors in the absence of retrograde hCDNF transport itself. Importantly, our results also indicate that the therapeutic effect of rhCDNF may be greater in mild to patients with moderate PD (Hoehn and Yahr stage 1–3), while in patients with more severe PD (Hoehn and Yahr stage >3), the efficacy may be reduced due to a low level of remaining DA neurons and axons (Hoehn and Yahr 1998; Cheng et al., 2010).

In conclusion, we show that rhCDNF spreads widely and relatively rapidly upon delivery and is cleared completely within a couple of days, having a half-life of 5.5 h. The exogenous rhCDNF is taken up by healthy striatal and cortical neurons via the common endocytotic pathway. Part of it might escape intracellular degradation, but the possible amounts of rhCDNF in organelles other than endocytic vesicles were below the detection threshold for immunoelectron microscopy. Finally, we show here for the first time that rhCDNF is retrogradely transported by DA neurons of the SNc, the neurons whose function rhCDNF preserves and restores in animal models of PD.

## Synthesis

Reviewing Editor: Leonidas Stefanis, Biomedical Research Foundation of the Academy of Athens Decisions are customarily a result of the Reviewing Editor and the peer reviewers coming together and discussing their recommendations until a consensus is reached. When revisions are invited, a fact-based synthesis statement explaining their decision and outlining what is needed to prepare a revision will be listed below.

The authors have responded in a satisfactory fashion to the points that were raised.
